# Infants’ Temperament and Mothers’, and Fathers’ Depression Predict Infants’ Attention to Objects Paired with Emotional Faces

**DOI:** 10.1007/s10802-015-0085-9

**Published:** 2015-10-08

**Authors:** Evin Aktar, Dorothy J. Mandell, Wieke de Vente, Mirjana Majdandžić, Maartje E. J. Raijmakers, Susan M. Bögels

**Affiliations:** Amsterdam Brain and Cognition, University of Amsterdam, Nieuwe Achtergracht 129, 1018WS Amsterdam, Netherlands; Research Institute of Child Development and Education, University of Amsterdam, Nieuwe Achtergracht 127, 1018WS Amsterdam, Netherlands; Department of Developmental Psychology, University of Amsterdam, Weesperplein 4, 1018XA Amsterdam, Netherlands

**Keywords:** Pupil dilation, Infancy, Emotion, Temperament, Fathers, Individual differences

## Abstract

Between 10 and 14 months, infants gain the ability to learn about unfamiliar stimuli by observing others’ emotional reactions to those stimuli, so called social referencing (SR). Joint processing of emotion and head/gaze direction is essential for SR. This study tested emotion and head/gaze direction effects on infants’ attention via pupillometry in the period following the emergence of SR. Pupil responses of 14-to-17-month-old infants (*N* = 57) were measured during computerized presentations of unfamiliar objects alone, before-and-after being paired with emotional (happy, sad, fearful vs. neutral) faces gazing towards (vs. away) from objects. Additionally, the associations of infants’ temperament, and parents’ negative affect/depression/anxiety with infants’ pupil responses were explored. Both mothers and fathers of participating infants completed questionnaires about their negative affect, depression and anxiety symptoms and their infants’ negative temperament. Infants allocated more attention (larger pupils) to negative vs. neutral faces when the faces were presented alone, while they allocated less attention to objects paired with emotional vs. neutral faces independent of head/gaze direction. Sad (but not fearful) temperament predicted more attention to emotional faces. Infants’ sad temperament moderated the associations of mothers’ depression (but not anxiety) with infants’ attention to objects. Maternal depression predicted more attention to objects paired with emotional expressions in infants low in sad temperament, while it predicted less attention in infants high in sad temperament. Fathers’ depression (but not anxiety) predicted more attention to objects paired with emotional expressions independent of infants’ temperament. We conclude that infants’ own temperamental dispositions for sadness, and their exposure to mothers’ and fathers’ depressed moods may influence infants’ attention to emotion-object associations in social learning contexts.

Learning about dangers in the environment via observing others’ reactions, so-called observational learning, vicarious learning or modeling, is a highly adaptive strategy that tremendously increases one’s chances for survival (Olsson and Phelps 2007). This is especially true for preverbal infants who know little about the dangers in the environment when they start to actively explore it following locomotion (Campos et al. 2000). The first forms of observational learning emerge between 10 and 14 months when infants start to actively use adults’ emotional expressions to guide their behavioral reactions to ambiguous/novel stimuli in the environment, so-called social referencing (SR; Emde 1992; Feinman 1982; Feinman, Roberts, Hsieh, Sawyer, and Swanson 1992). Observational SR studies where infants are confronted with ambiguous stimuli like strangers or robot toys have consistently revealed that infants are less likely to interact with ambiguous/novel stimuli, and more likely to manifest negative affect (i.e., fear) and avoidance when the referee expresses negative as compared to positive and neutral emotions (Feinman et al. 1992; Vaish et al. 2008). The present study investigated infants’ attention to emotion and referential cues (i.e., head and gaze direction) as essential components of SR processes, and explored for the first time infants’ negative temperament, parents’ negative affect, depression and anxiety as potential sources of individual differences in infants’ attention in triadic emotion learning/SR contexts.

## Infants’ Temperament and Exposure to Parental Depression and Anxiety as Sources of Individual Differences in Infants’ Behavioral Reactions to Novelty in SR Situations

Infants’ biases towards negative emotional signals in SR situations are adaptive in typical development, while repeated exposure to high fear and anxiety expressions from parents with anxiety disorders in SR situations seem to constitute risk for parent-to-infant transmission of anxiety in infants with negative temperamental dispositions (Aktar, Majdandžić, De Vente, and Bögels 2013; Murray et al. 2008). Infants’ temperamental dispositions refer to individual differences -of biological origin- on reactivity and self-regulation in affect, attention and behavior in the face of novelty (Rothbart 2007; Rothbart and Bates 2006). Negative temperamental dispositions, characterized by fearful, distressed and/or avoidant reactions to novelty/ambiguity are considered as a general vulnerability to the effects of adverse rearing environments (Ingram and Luxton 2005; Nigg 2006). Negative temperamental dispositions are more common in children of parents with depression and anxiety as compared to children of parents without psychopathology, and are linked to higher risk for later depression and anxiety (Biederman, Rosenbaum, Chaloff, and Kagan 1995; Bruder-Costello et al. 2007; Rosenbaum et al. 1993). Temperamentally fearful infants of parents (with and without anxiety diagnosis) were found to show more avoidance of ambiguous stimuli when exposed to more anxious reactions from parents in SR situations (Aktar et al. 2013; De Rosnay, Cooper, Tsigaras, and Murray 2006). Moreover, exposure to high levels of anxiety from parents with anxiety diagnoses were shown to be linked to a longitudinal increase in temperamentally fearful infants’ avoidance of ambiguous stimuli in SR situations (Murray et al. 2008). Exposure to parents’ expressions of anxiety in the face of anxiety-provoking stimuli in SR is therefore assumed to be an early mechanism in early modeling of fears, and an early pathway for parent-to-infant transmission of anxiety for temperamentally fearful infants (Murray et al. 2008; Murray, Creswell, and Cooper 2009). In contrast to anxiety, the effect of exposure to parents’ depression has not yet been investigated in SR situations.

Considering high prevalence of depression and anxiety disorders in parents in the postnatal year (O’Hara and Swain 1996; Matthey, Barnett, Howie, and Kavanagh 2003; Ross and McLean 2006) and significant associations between early exposure to parental depression and anxiety and offsprings’ later behavioral, emotional and psychological functioning (e.g., Avan, Richter, Ramchandani, Norris, and Stein 2010; Murray et al. 1999; Pawlby et al. 2008), it is important to better understand how exposure to parental negative emotions in SR situations in the case of parental depression and anxiety affects infants’ reactions. From a developmental psychopathology perspective, a better understanding of the effects of exposure to parents’ negative emotions in infancy requires the consideration of continuities and discontinuities between normal vs. clinical parent samples in the effects of exposure to parental negative emotions on infants’ reactions to ambiguous stimuli (Cicchetti 2006). Thus, investigating the non-clinical variation in parents’ expressions of negative emotions, depression and anxiety as a predictor of infants’ reactions in SR contexts are highly informative in understanding the effects of exposure to parental expressions in clinical samples.

### Separating Social Referencing into its Components

SR requires infants to attend to the adults’ emotional signals during person-infant-object interactions, to link the emotional signals to the stimulus (emotion-object associations), and to regulate their behavior accordingly (Feinman et al. 1992). Among these three essential components of social referencing skills, previous SR studies have predominantly relied on naturalistic observations of infants’ regulation of behavioral and emotional reactions (i.e., the third component of social referencing). This research has greatly advanced our understanding of infants’ emotion and behavior regulations in everyday SR situations. Scientific interest has recently grown on the investigation of neural and physiological correlates of infants’ ability to form associations between the emotional signal and the stimulus (i.e., the second component) in SR situations. To form the emotion-object associations, infants have to jointly process the threat value of referents’ emotional expressions and the referential cues (i.e., head and gaze direction) that link referents’ expressions to the ambiguous stimulus (Feinman et al. 1992). A previous study by Moses, Baldwin, Rosicky, and Tidball (2001) has revealed that infants rely on adults’ head and gaze direction in SR situations to infer whether an emotional signal relates to the ambiguous stimulus. In the current study, we used a physiological index of attention allocation to investigate how emotion-object associations alter infants’ attention to unfamiliar objects. Our goal was to test the effects of emotion and gaze direction as essential components of SR skills on infants’ attention in triadic person-infant-object contexts. Furthermore, we aimed to explore whether negative temperamental dispositions of typically developing infants and parents’ negative affect, depression and anxiety explain individual differences in infants’ attention allocation (measured via pupillary responses) to objects paired with emotional versus neutral facial expressions gazing towards vs. away from the objects.

### Investigating Infants’ Attention to Emotion-Object Associations in SR contexts

Infants do not need to be active participants in triadic person-infant-object interactions to form emotion-object associations. A study by Mumme and Fernald (2003) revealed that SR processes can be activated by computerized presentations of emotion-object associations in SR. In this study, 12-month-old infants were first presented with actresses expressing positive and negative (vs. neutral) towards unfamiliar objects. Following the presentation of emotion-object associations, infants were presented with the objects (previously presented on the screen) in real life for 30s. In line with the findings from naturalistic interactions, infants were found to interact less with the objects, and to express more negative affect when they have previously seen the objects paired with the actress expressing negative (vs. neutral) emotions. Another study using event-related potentials (ERP) as a physiological index of attention revealed an increase in neural correlates of 12-month-old infants’ attention to novel objects after these have been paired with caregivers’ facial and vocal expressions of negative (but not positive, vs. neutral) expressions (Carver and Vaccaro 2007). Moreover, this study revealed positive associations between ERP correlates of infants’ attention to novel objects and their behavioral reactions during the emotion-object pairings (i.e., Negative Central [Nc] component and observed interest and proximity to the toys). The use of computerized tasks to investigate SR processes enables the investigation of infants’ attention allocation as a potential mechanism that may explain the behavioral findings revealing high reactivity to novelty in SR situations.

A series of studies testing the effects of emotion and gaze direction in younger (3–to–7-month-old) infants revealed that infants start to allocate enhanced attention to negative expressions gazing towards objects in the physiological level before SR processes fully manifest at the behavioral level (e.g., Hoehl and Striano 2010b). Using ERP correlates (i.e., Nc), Hoehl and colleagues assessed younger infants’ attention in triadic (person-infant-object) contexts via fully computerized tasks of SR to investigate the role of emotion and gaze/head direction as early antecedents of SR processes (e.g., Hoehl, Palumbo, Heinisch, & Striano 2008a; Hoehl and Striano 2010a, 2010b).

In this paradigm, infants’ attention was measured during the pairing of novel objects with emotional (vs. neutral) facial expressions gazing towards (vs. away from) these objects (e.g., Hoehl et al. 2008a; Hoehl and Striano 2010a) and during the presentation of objects alone following the face-object pairing (e.g., Hoehl and Striano 2010b; Hoehl et al. 2008b). These studies have shown that 6 and 7-month-olds allocate more attention to fearful (vs. neutral) faces only when the gaze is directed towards (vs. away from) the objects during the emotion-object pairing (Hoehl et al. 2008a; Hoehl and Striano 2010b). Furthermore, 3 and 6-month-old infants allocate more attention to the objects following the pairing of these with fearful (vs. neutral) faces gazing towards the object (Hoehl and Striano 2010b; Hoehl et al. 2008b). In contrast, when the gaze is directed away from the object, no difference was found in infants’ attention allocation to objects during and following the pairing of these objects with fearful vs. neutral faces. Thus, fearful faces seem to elicit enhanced attention only in contexts where gaze direction helps the infant to disambiguate/clarify the referent of the threat signal. However, a different pattern of results was reported at 9 months (Hoehl and Striano 2010b), where both SR skills and negativity biases are about to emerge in infants’ behavior (Vaish et al. 2008; Walden and Ogan 1988). Nine-month-olds allocated more attention to fearful vs. neutral faces paired with objects independent of gaze direction, while they showed more attention to objects that have been previously paired with neutral vs. fearful faces gazing towards objects. It remains unclear why infants would show this different pattern of responses at 9 months, right before the emergence of SR processes.

In contrast to previous evidence that predominantly focused in the first three quarters of the first year, infants’ processing of emotion and referential cues during emotion-object associations has not been studied during the developmental period when all infants actively use SR. Our focus in the current study was on infants’ attention allocation at the age following the emergence of SR skills (after 14 months; Walden and Ogan 1988). Using an eye-tracking adaptation and extension of the experiments by Hoehl and colleagues (e.g., Hoehl and Striano 2010b) we tested how 14–to–17-month-old (*range =* 14.39 to 16.69) infants’ attention to unfamiliar objects changes after these have been paired with emotional (vs. neutral) facial expressions gazing towards (vs. away from) the objects. Three additional issues in infants’ joint processing of gaze and emotion summarized below were explored in the current study.

### The Effect of Emotion on Infants’ Attention: Valence and Threat Relevance

Because existing evidence on emotion and gaze processing is predominantly on the comparison of fearful and neutral emotions (Hoehl et al. 2008a, 2008b; Hoehl and Striano 2010b), it remains largely unknown whether the attention enhancing effect of fearful facial expressions also holds for other positive or negative facial expressions. Given the survival value of SR skills and negativity bias in infancy, one would expect this effect to be especially salient for negative emotions with threat relevance (i.e., fear and anger) as compared to positive emotions. In a previous comparison of happy (vs. neutral) faces on 3-month-olds’ processing of novel objects in the same SR paradigm, Hoehl and Striano (2010a) found that infants showed enhanced attention to the presentation of happy faces gazing towards the objects. However, this pairing did not alter infants’ attention allocation when these objects were later presented alone. These findings reveal that the attention enhancing effect of fearful faces in triadic contexts may not apply to positive emotions. However, it is still unclear whether the differences between these later findings with happy faces and earlier findings with fearful faces (Hoehl et al. 2008a, 2008b; Hoehl and Striano 2010b) are due to differences in emotional valence (positive vs. negative) or in threat relevance of these emotions. To address this, we investigated how infants’ attention to unfamiliar objects changes after the objects were paired with threat-relevant (i.e., fear) or not threat-relevant (i.e., sad) negative emotional expressions, and with positive emotional expressions (i.e., happy) as compared to neutral facial expressions.

### Measurement of Infants’ Attention

Existing physiological evidence on the effects of emotion and gaze direction on infants’ attention comes from face-sensitive components of ERP responses. In recent years, pupil dilation has provided a promising alternative to ERP as a physiological index of attention allocation in emotion research in infancy (Geangu, Hauf, Bhardwaj, and Bentz 2011; Gredebäck, Eriksson, Schmitow, Laeng, and Stenberg 2012). Pupillary responses are mediated through brain structures involved in quick processing of biologically relevant stimuli like faces (like the amygdala and the locus coeruleus; Aston-Jones and Cohen 2005; Aston-Jones, Rajkowski, Kubiak, Valentino, and Shipley 1996), and in stress responses to threat (Lipski 2012). Pupil dilation in response to affectively loaded stimuli under uniform lighting conditions has been found to reflect sympathetic processing, and attention allocation in adults (Bradley, Miccoli, Escrig, and Lang 2008). Findings from recent pupil studies in infants revealed increased pupil dilation to negative as compared to neutral emotions (Geangu et al. 2011; Gredebäck et al. 2012). To our knowledge, no studies so far measured the joint effects of emotion and referential cues on infants’ attention via pupil dilation in triadic (person-infant-object) contexts.

### Infants’ Temperament and Exposure to Parental Depression and Anxiety As Sources of Individual Differences in Infants’ Attention to Novel Objects

Individual differences in infants’ attention to gaze and emotional expressions have not yet been investigated in computerized SR experiments. In the current study, we tested whether infants’ temperament and parents’ negative affect, depression and anxiety explain individual differences in infants’ attention to faces, and to objects that were paired with these faces. Like summarized above, behavioral evidence (Aktar et al. 2013; De Rosnay et al. 2006) has consistently revealed a positive association between parents’ expressions of anxiety in SR situations and infants’ avoidance of novel stimuli for temperamentally fearful infants of parents with and without anxiety disorders. More recent investigations of the effects of temperament and of exposure to parents’ negative emotions, depression and anxiety on infants’ attention in computerized emotion experiments illustrate the relevance of considering intrapersonal and interpersonal processes in infants’ attention allocation. For example, available neurophysiological evidence on infants’ attention allocation to facial expressions reveals significant associations between 3–to–13-month-old infants’ fearful temperament and the ERP indices of their attention allocation to fearful and happy faces (De Haan, Belsky, Reid, Volein, and Johnson 2004; Martinos, Matheson, and De Haan 2012). Moreover, a negative association between temperamentally positive infants’ attention to fearful faces and their exposure to parents’ positive (but not negative) affect was found in typically developing infants (De Haan et al. 2004). Likewise, studies focusing on emotion processing among infants of clinically depressed (vs. non-depressed) mothers have revealed a decrease in infants’ attention to sad facial expressions when the mother is clinically depressed (Field et al. 1998). Taken together, these findings provide preliminary support for the idea that infants’ negative temperament and their exposure to parents’ negative expressions are important correlates of infants’ attention allocation to negative facial expressions and physiological reactivity to ambiguous stimuli at the end of first year, while the direction of the associations remains to be further investigated. Considering that information processing in the case of depression and anxiety disorders is characterized by enhanced attention/vigilance to negative emotion (Leppänen 2006; Van Bockstaele et al. 2014), physiological indices of infants’ attention allocation may constitute an important outcome in infancy that may be useful in detecting early risk for psychopathology.

### Aims and Hypotheses

To summarize, this study aimed to investigate infants’ attention to emotion and referential cues as essential components of emotion-object associations in SR and to explore infants’ negative temperament as well as parents’ negative affect, depression and anxiety as potential sources of individual differences in infants’ attention in triadic emotion learning contexts. Infants’ pupil diameters were recorded during a computer task where unfamiliar objects were presented first alone, then paired with a facial expression with referential cues of head/gaze direction, and then alone again. The effects of emotion and head/gaze direction were investigated on infants’ pupillary responses to facial expressions and novel objects paired with these expressions in a sample of typically developing infants between 14 and 17 months of age. Infants’ negative temperament as well as parents’ negative affect, depression and anxiety were measured with questionnaires filled in by both the mother and father of each participating infant.

Based on previous evidence on infants’ pupil dilation to emotion (e.g., Geangu et al. 2011; Gredebäck et al. 2012), we predicted a significant effect of emotion on infants’ pupil dilation, such that infants’ should show increased attention (larger pupils) to emotional as compared to neutral facial expressions. Further, if the effect extends to object paired with faces, then infants should have a more pronounced increase in pupil size for objects that were paired with emotional (vs. neutral) faces. Additionally, we explored differences in pupil responses to different emotional expressions. If the effect of emotion is specific to negative emotions, infants should respond with increased attention (larger pupils) to both sad and fearful faces but not to happy and neutral ones. Additionally this increased attention should also extend/generalize to the objects that were paired with these faces. In contrast, if the effect of emotion is specific to threat-relevant emotions, infants should allocate increased attention to fearful, but not to sad or happy faces (and to objects paired with these faces). The effect of referential cues (gaze direction), which was found to affect infants’ attention in previous ERP studies, was explored for the first time using pupil responses. If infants are sensitive to referential cues, there should be increased attention (larger pupils) to objects that are paired with faces gazing towards (vs. away from) the objects.

In the light of previous behavioral (Aktar et al. 2013; De Rosnay et al. 2006), and ERP evidence (De Haan et al. 2004), we expected that infants’ negative temperament as well as parents’ negative affect, depression and anxiety explain individual differences in infants’ attention to emotion in triadic emotion learning contexts. In view of previous evidence revealing a moderating role of infants’ temperament on the physiological correlates of infants’ attention (De Haan et al. 2004), we also explored how the associations of parents’ negative affect, depression and anxiety with infants’ pupil responses to emotion change as a function of infants’ temperament.

## Methods

### Participants

The sample for the study consisted of 57 infants (31 girls, *M*_*Age*_ = 15.25 months, *SD* = 0.48*, range =* 14.23 to 16.69 months), of which 43 infants visited with their mother, 10 with their father, and 4 with both parents. An additional 13 participants were tested but removed from analysis (due to fussiness, equipment failure and missing data, see *Data Reduction*). Of 57 infants who participated, the questionnaire data was partially or fully available from 54 mothers and 48 fathers. (see *Results* for more information about missing scores per questionnaire). Families were part of a larger sample recruited via invitation letters sent by the municipality to families who recently became parents. Sociodemographic characteristics are presented in Table 1. The study was approved by the ethics committee at the University of Amsterdam. Parents provided informed consent for participation.

**Table 1:** Sample characteristics

		Mother	Father
Parents’ age	*M* (*SD*)	34.35 (4.19)	37.43 (5.16)
	*N*	54	47
	*Range*	27–46	30–52
Dutch origin	%	79.63	95.75
	*N*	54	47
Educational level	Secondary education (high school) %	3.70	14.89
	Professional education %	3.70	6.38
	Higher professional education %	16.67	27.66
	University %	70.37	51.06
Professional level	Unemployed %	–	–
	Employed %	85.19	89.36
	Self -employed %	14.82	10.64
Professional status	House Keeper %	3.70	–
	Part Time %	64.82	21.74
	Full Time %	25.93	78.26
Monthly income^a^	*M (SD)*	4.43 (1.76)	5.41 (1.44)
	*N*	49	44
	*Range*	1–7	3–7
Working hours (per week)	*M (SD)*	30.24 (9.32)	37.80 (8.15)
	*N*	51	46
	*Range*	0–52	15–60

^a^ Parental income was assessed with a 7-point scale from 1 (<500 euros/month) to 7 (>5000 euros/month)

### Materials and Procedure

#### Stimuli

Stimuli were colored photographs of 16 unfamiliar objects (from the Novel Object and Unusual Name Database; NOUN; Horst & Hout 2015). Because infants are known to be sensitive to the incongruent emotion-object associations towards the end of first year (Hirshberg and Svejda 1990), we used pictures of different objects in each trial, instead of using the same objects with both positive and negative faces. The stimulus set had widths varying from 3.19 to 7.64 cm, *M* = 5.23, *SD* = 1.34; and heights varying from 4.77 to 8.72 cm, *M* = 6.78, *SD* = 1.11. All objects were presented on a black background, alone or together with portrait photographs of a female model exhibiting neutral, fearful, happy, and sad facial expressions (9 cm × 15 cm). Considering the task demands, we chose to use the facial expressions of a single model in the current study. The photographs were taken from the Radboud Faces Database (Langner et al. 2010). Head/gaze direction of the actor was directed either towards or away from the objects. One randomly determined combination of objects and emotional facial expressions was used for all infants in the experiment.

#### Procedure

Infants’ pupil diameters were measured during the task via a Tobii T120 eye-tracker in a dimly lit room. Infants were secured in a car seat that was situated 60 cm away from the screen. The parent sat on a chair behind the infant during the tasks and was instructed not to intervene unless the infant became fussy.

**Fig. 1:**
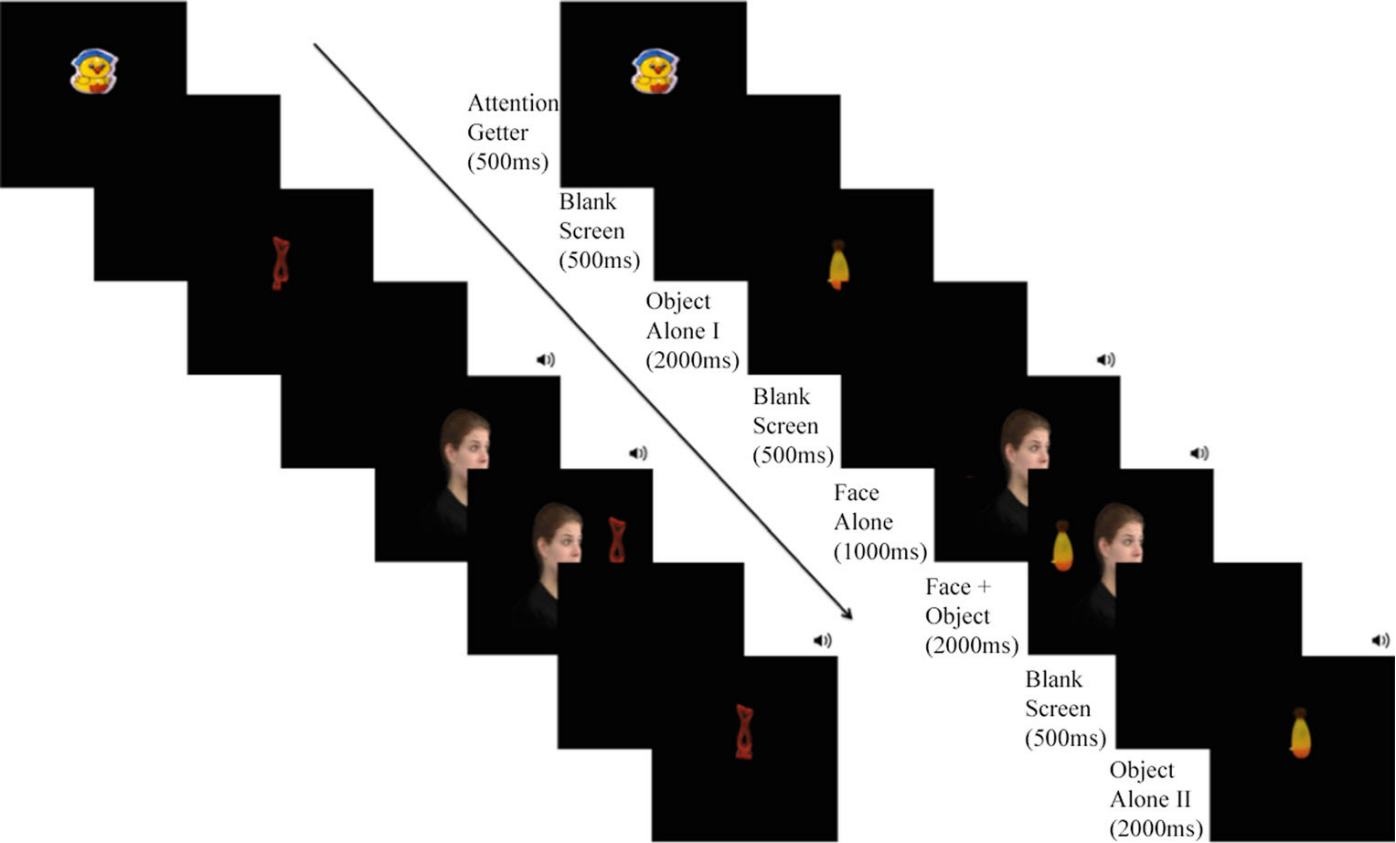
Time flow of trials This figure illustrates the time flow of two consecutive trials with fearful expressions. The trials in each block were clustered in pairs, such that each emotional expression with one head/gaze direction (fearful faces with the right head/gaze direction in the figure) appeared in two consecutive trials with two different objects on the left and right side of the screen. The order of the objects’ position in each pair of trials (presented on the right and left side of the figure) was randomly determined. The same expression appeared with the reverse head/gaze (i.e., fearful faces with left head/gaze direction in this case) and with novel objects in the other block. Each trial started with an attention getter displayed at the center for 500 ms, and consisted of object presented at the center of the screen (Object Alone I), a face appearing at the center of the screen with head and gaze towards the left or right, first alone (Face Alone), and then together with the object on the left or right side of the screen (Object + Face). The trial ended with the second presentation of the object alone at the center (Object Alone II). A 70 ms auditory sound was used as attention getter at time points indicated with a sound icon.

The experiment consisted of a total of 16 trials that included 4 facial expressions with 2 head/gaze directions (left vs. right), and 2 object positions (on the left vs. right of the screen; see Fig. 1). There were 2 blocks of 8 trials. Each block started with the neutral face-object pair and continued in a randomly generated order of (non-neutral) emotions. Two trials from a given emotional expression with a given head/gaze direction (for example, fearful faces with the right head/gaze direction) appeared in two consecutive trials where two different objects randomly appeared either on the left or on the right side of the screen, forming a pair of trials. An example of a pair of trials is presented in Fig. 1. Each emotional expression was clustered in one pair of trials in each block. The same expression appeared in the second block with the reverse head/gaze direction (for example, fearful faces with the left head/gaze direction) and with novel objects. The order of the objects’ position (left, or right) in each pair of trials per block, and the order of emotion following neutral trials were randomly determined in each block.

Each trial started with an attention getter displayed at the center for 500 ms, and consisted of the following events (see Fig. 1): Colored object presented at the center of the screen (Object Alone I; 2000 ms), a face appearing at the center of the screen with head and gaze towards the left or right, first alone (Face Alone; 1000 ms), and then together with the object on the left or right side of the screen (Object + Face, 2000 ms). The trial ended with the second presentation of the object alone at the center (Object Alone II; 2000 ms). A 70 ms sound was presented together with a blank screen after the first presentation and before the second presentation of the object, and without a blank screen between the presentations of Face Alone and Object + Face to attract infants’ attention. Prior to the start of each trial, an experimenter monitored infants’ attention and repeated the presentation of the visual attention getters when necessary.

### Questionnaires

#### Infants’ Temperament

To measure infants’ temperamental dispositions for negative emotions, the mother and father of each participating infant were asked to fill out the fear and sadness scales of the Infant Behavior Questionnaire Revised (IBQ-R; Gartstein and Rothbart 2003) where parents rate the frequency of infants’ expressions of fear (such as crying, or showing distress) and sadness (e.g., becoming tearful and sad) in number of occasions (e.g., fear while visiting a new place or meeting a stranger, or sadness after separation from the caregiver) during the most recent 2 weeks on a 7-point scale. Out of 57 couples that participated with their infant, the data was available from 53 mothers and 43 fathers for infants’ fearful temperament and from 54 mothers and 46 fathers for infants’ sad temperament. The reliability (*Cronbach’s α*, for mothers and fathers, respectively) was 0.92 and 0.92 for fear, and 0.76 and 0.84 for sadness. The correlations between mothers’ and fathers’ ratings of infant temperament was *r* = 0.56, *p* < 0.001 for fear and *r* = 0.40, *p* = 0.006 for sadness. The mother’s and the father’s ratings on each scale were averaged to calculate infants’ fear and sadness scores.

#### Parents’ Negative Affect

To measure parents’ negative affect, we asked both parents to fill in the Negative Affect Schedule of The Positive and Negative Affect Schedule (PANAS; Watson, Clark, and Tellegen 1988). The Negative Affect Schedule (NAS) consists of 10 negative emotions, and measures on a 5-point scale the extent to which parents experienced these emotions in the last 2 weeks. Out of 57 couples that participated with their infant, the data was available from 54 mothers and 47 fathers for the NAS. The reliability of the scale (*Cronbach’s α*) was 0.79 for mothers and 0.87 for fathers, respectively. The correlation between mothers’ and fathers’ negative affect was not significant (see Table 2).

**Table 2:** Pearson correlations between infant temperament, parental negative affect, depression and anxiety

		Infant fearful temperament	Infant sad temperament	Maternal negative affect	Maternal depression	Maternal anxiety	Paternal negative affect	Paternal depression
Infant fearful temperament	*r*							
	*n*							
Infant sad temperament	*r*	0.34*						
	*n*	53						
Maternal negative affect	*r*	0.04	0.08					
	*n*	53	53					
Maternal depression	*r*	0.08	0.00	0.50**				
	*n*	53	53	54				
Maternal anxiety	*r*	0.27	0.14	0.42**	0.73**			
	*n*	53	53	54	54			
Paternal negative affect	*r*	–0.05	0.18	0.04	0.10	0.09		
	*n*	47	47	47	47	47		
Paternal depression	*r*	–0.02	0.03	–0.18	0.13	0.20	0.38*	
	*n*	44	44	44	44	44	44	
Paternal anxiety	*r*	–0.08	0.29	0.01	–0.06	0.15	0.45**	0.40**
	*n*	44	44	44	44	44	44	44

*. Correlation is significant at the 0.05 level (2-tailed)

**. Correlation is significant at the 0.01 level (2-tailed)

#### Parents’ Depression and Anxiety

To measure parents’ symptoms of depression and anxiety, we asked both parents to fill in the second edition of Beck Depression Inventory (BDI-II; Beck, Steer, and Brown 1996), a 21-item questionnaire measuring depressive symptoms, as well as the adult version of Screening for Anxiety Related Emotional Disorders (SCARED-A; Bögels and Van Melick 2004), a 71-item questionnaire assessing symptoms of anxiety disorders. Out of 57 couples that participated with their infant, the data was available from 54 mothers and 44 fathers for these questionnaires. In the current study, the reliability of BDI-II was (*Cronbach’s α*) 0.86 for mothers and 0.78 for fathers, and of SCARED-A, 0.89 for mothers and 0.88 for fathers. The correlations between parents’ scores on depression and anxiety as well as negative affect are presented in Table 2.

### Statistical Analyses

#### Data Reduction

The following steps were carried out to obtain the outcome variables: First, outlying values (>4SDs) in each infants’ pupil diameters were removed. Second, missing observations (<500 ms) were replaced via linear interpolation (see Jackson and Sirois 2009) to account for blinks and tracking errors following the exclusion of outlying cases in the first step. Third, the pupil data was reduced to observations where the infant looked at the object or face, and was aggregated to 50 ms time intervals.

#### Outcome Variables

Two main outcome measures, obtained from the presentations of Face Alone and Object Alone were used in the analyses. The first outcome consisted of infants’ pupil diameters to the 1000 ms presentation of Face Alone (20 observations with 50 ms intervals), averaged across left and right eyes. This outcome was used to measure infants’ attention to facial expressions, when presented without an object. Trials in which children looked at the presentation of Face Alone for less than 500 ms were excluded from analyses of facial expressions the dataset (see Gredebäck et al. 2012). The second outcome concerned the change in infants’ pupil responses to objects alone after the pairing of these with faces in the Object + Face presentation. To analyze how infants’ processing of the objects changes after being paired with faces, we subtracted the pupil diameters during the first presentation of the object from the pupil diameters in the second presentation of the object (Object alone II – Object alone I) from the left and the right eye at each 50 ms step of the 2000 ms presentation. The difference scores obtained from the right and left pupils were then averaged at each of the 40 observation points (50 ms steps of the 2000 ms presentation of Object Alone). Trials in which children looked at the presentation of Object + Face Presentation for less than 500 ms were excluded from analyses of object processing because it was unclear whether they had enough time to process emotion-object association. Due to limited attention span in infancy, we chose not to apply further restrictions concerning the minimum number of trials that each infant must complete to be included. We adjusted our analytic approach accordingly and used multilevel models that are known to accommodate missing data (Bagiella, Sloan, and Heitjan 2000). Of 57 infants who generated the data for the analyses, the eye-tracking data was available from 41 infants for Face Alone, and 56 infants for Object Alone Trials. Infants contributed to the data in average with 8.73 trials for the analyses of Object Alone (*SD* = 4.63, *range:* 1 to 16) and with 6.90 trials for the analyses of Face-Alone (*SD* = 5.18*, range*: 1 to 16). The number of available trials was not significantly associated with infants’ temperament, or with parents’ negative affect, depression or anxiety scores.

#### Main Analyses

Both outcomes were analyzed with multilevel regression models using auto-regressive covariance structure for repeated observations of pupil responses. Repeated observations of pupil response within and across trials were nested within infants. The repeated observations within trial consisted of infants’ pupil dilation to the presentations of Face Alone or Object Alone in 50 ms intervals during 1000 ms time window of Face Alone presentation or 2000 ms time window of Object Alone presentation. The repeated observations of pupil dilation between trials consisted of 16 repetitions of Face Alone and Object Alone presentations. The intercept and picture time were random effects in both models. Emotion (i.e., type of facial expression) and gaze (i.e., gaze direction) effects were treated as fixed effects, along with other predictors. Neutral expression and gaze/head towards object were the reference for emotion and referential cues in the regressions. Infants’ temperament, and parents’ negative affect, anxiety and depression were entered as continuous predictors in the models. Inspection of distributions indicated sufficient normality; skewness and kurtosis of all variables were < |2|, except for maternal anxiety. Three mothers with outlying scores (>3 SDs) of anxiety were replaced by the next most extreme value in the distribution. To control for order effects, order of trials was included as a continuous variable in both models. Mean luminance (*M* = 12.14, *SD* = 0.77, *range*: 11.08 to 13.15) of each face was additionally included in the analysis of infants’ pupil responses to faces as a control variable. Scores on outcome variables and on maternal negative affect, anxiety, and/or depression were standardized for the analyses. The raw correlations between predictor variables are presented in Table 2.

The initial multilevel model for infants’ processing of facial expressions (the face model) consisted of the main effects of emotion, picture time, order and luminance. The initial multilevel model for infants’ processing of objects (the object model) consisted of the main effects of emotion, picture time, gaze direction, and order. The interaction between emotion and gaze direction was tested in the object model in the next step. Although the main effect of infants’ gender and its two-way interactions with emotion were initially added as additional predictors of infants’ pupil responses to faces, and to objects paired with faces in the analyses, none of these effects were significant in the current models. Infants’ gender was therefore removed from the analyses.

To investigate individual differences in infants’ pupil responses, first the main effects of infants’ fearful and sad temperament and of mothers’ and fathers’ negative affect were included in both models. Second, two-way interactions of these predictor variables with emotion were included. In the next step, the two-way interactions between parents’ negative affect and infants’ temperament were tested.

Finally, we explored the associations of infants’ pupil responses with parental depression and anxiety symptoms by separately analyzing these predictors in additional models. We repeated the same multilevel analyses, and tested the same interactions as the individual difference models described above. We first tested a model for parental depression (including infant temperament, excluding parental negative affect), and then a model for parental anxiety symptoms. Interactions tested in each step were included in or removed from the models based on *t*-tests. All the effects were evaluated at *α* = 0.05. To probe significant interactions, we used online tools by Preacher, Curran, and Bauer (2006, http://www.quantpsy.org/interact/hlm2.htm). We first inspected the 95 % confidence bands representing continuously plotted confidence intervals for the outcome slope across levels of the moderator. When the confidence interval does not include y = 0 for a given level of the moderator, the effect is interpreted as being significant at that level (i.e., *p* ≤ 0.05). Next, we plotted the association of the predictor with the outcome across low, moderate and high levels of the moderator. Moderate, low and high levels were the mean, 1 *SD* below and 1 *SD* above the mean, respectively. Because confidence intervals cannot be computed for two-way interactions in the case of dichotomous variables (e.g., emotion) we based our interpretations on the interaction plots only in these cases.

## Results

### The Effect of Emotion on Infants’ Attention to Faces

The initial face model for infants’ processing of facial expressions is presented in Table 3 (*n =* 41). The effect of emotion and of picture time was significant in this model. Infants’ pupils were more dilated for negative (fearful and sad) vs. neutral facial expressions, while there was no significant difference in infants’ pupil dilation to happy (vs. neutral) faces. Infants’ pupil reactivity decreased over (picture) time.

**Table 3:** The effect of emotion on infants’ pupil dilation to faces (3.a), to objects paired with faces (3.b)

3.a				3.b			
Parameter	*B*	*SE*	*p*	Parameter	*B*	*SE*	*p*
Intercept	−0.14	0.42	0.732	Intercept	0.44	0.12	< 0.001
Happy	0.15	0.10	0.117	Happy	−0.45	0.15	0.003
Fearful	0.33	0.09	< 0.001	Fearful	−0.40	0.15	0.007
Sad	0.22	0.09	0.016	Sad	−0.46	0.15	0.002
Picture time	−0.04	0.00	< 0.001	Head/gaze direction	0.00	0.08	0.978
Order	−0.01	0.02	0.356	Picture time	−0.01	0.00	0.001
Block	0.13	0.13	0.340	Order	0.00	0.03	0.946
Luminance	0.06	0.03	0.066	Block	−0.17	0.23	0.448

The neutral face was the reference for emotion effects. *R* ^*2*^ *=* 0.85 in 3.a and 0.35 in 3.b

### The Effect of Emotion and Referential Cues on the Change in Infants’ Attention to Objects

The initial object model for the change in infants’ processing of objects (*n* = 56) is presented in Table 3. The effect of emotion and of picture time was significant in this model, while the effect of head/gaze direction was not significant. The change in pupil dilation decreased over (picture) time. Infants showed smaller increases in their pupil dilation to objects paired with happy, fearful and sad faces, as compared to those paired with neutral faces. The interaction between head/gaze direction and emotion was not significant in this model.

### Individual Differences in Infants’ Attention to Faces

**Fig. 2:**
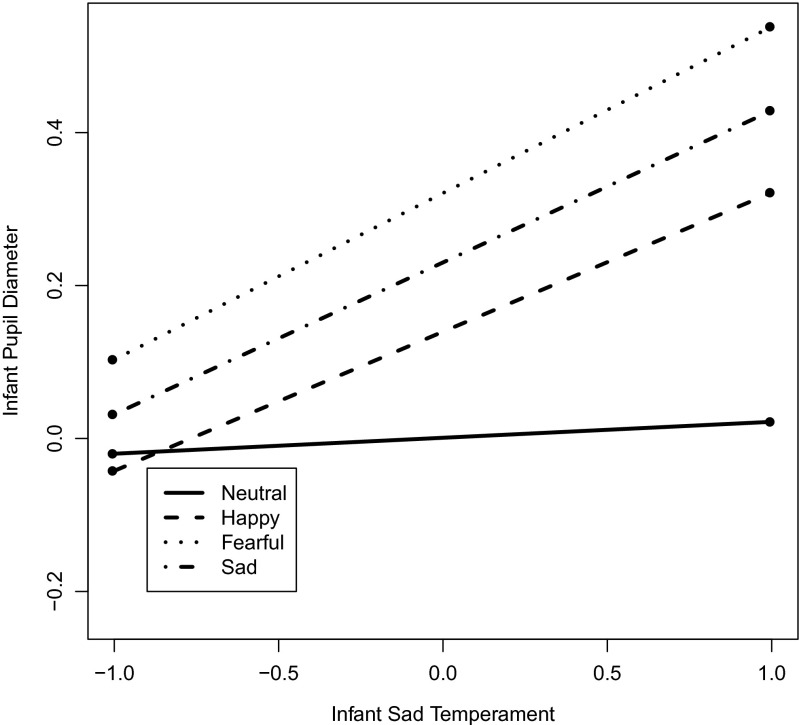
The plot for the association between infants’ sad temperament (z-scores on the x-axis) and their pupil response (z-scores on y-axis) to emotional facial expressions as a function of emotion. The association between infants’ pupil response and infants’ sad temperament was positive for fearful, happy and sad (vs. neutral) faces

To test individual differences in infants’ pupil responses, we first added the main effects of infants’ fearful and sad temperament, and of parents’ negative affect to the initial face model in Table 3. None of these effects were significant. Among tested interactions, only the interaction between infants’ sad temperament and emotion was significant and was kept in the final model, presented in Table 4 (*n* = 33^1^). Infants with higher scores on sadness allocated more attention to happy, fearful and sad faces (see Fig. 2). Neither the main effects, nor two-way interactions of parental depression and anxiety with emotion and infants’ temperament did significantly predict infants’ attention to facial expressions.

**Table 4:** The associations of infants’ pupil dilation to faces with infants’ negative temperament and with parental negative affect

Parameter	*B*	*SE*	*p*
Intercept	0.01	0.45	0.989
Happy	0.13	0.10	0.171
Fearful	0.31	0.09	0.001
Sad	0.22	0.09	0.014
Picture Time	−0.05	0.01	< 0.001
Infant Fear	0.02	0.22	0.930
Infant Sadness	0.03	0.23	0.912
Maternal Negative Affect	0.10	0.18	0.576
Paternal Negative Affect	−0.10	0.17	0.559
Happy*Infant Sadness	0.16	0.07	0.019
Fearful*Infant Sadness	0.19	0.07	0.004
Sad*Infant Sadness	0.17	0.06	0.008
Luminance	0.05	0.03	0.110
Order	−0.02	0.02	0.251
Block	0.17	0.13	0.205

The neutral face was the reference for emotion effects. *R* ^*2*^ = 0.87

Due to complications arising from hierarchical structure of multilevel regression models (Snijders and Bosker; 1994), and to different sample sizes in the models, R^2^ change cannot be interpreted as a relative goodness-of-fit measure across models

### Individual Differences in the Change in Infants’ Attention to Objects Following the Emotion-Object Pairing

To test individual differences in the change in infants’ processing of objects paired with facial expressions, we first added the main effects of infants’ temperament, and of parents’ negative affect to the object model in Table 3. This model is presented in Table 5 (*n* = 46^2^). Among main effects, the effect of maternal (but not paternal) negative affect was significant. Higher levels of maternal negative affect predicted a less pronounced difference in infants’ pupil responses to objects after the pairing with emotional (vs. neutral) facial expressions. None of the tested interactions were significant in this model.

**Table 5:** The associations of infants’ pupil dilation to objects with infants’ negative temperament and with parental negative affect

Parameter	*B*	*SE*	*p*
Intercept	0.42	0.13	0.002
Happy	−0.31	0.17	0.076
Fearful	−0.29	0.17	0.090
Sad	−0.36	0.17	0.033
Head/gaze direction	0.03	0.09	0.707
Picture time	−0.01	0.00	0.003
Infant fear	0.08	0.10	0.421
Infant sadness	0.09	0.10	0.374
Maternal negative affect	−0.20	0.09	0.037
Paternal negative affect	0.10	0.10	0.315
Order	−0.02	0.03	0.582
Block	−0.04	0.26	0.869

The neutral face was the reference for emotion effects. *R* ^*2*^ = 0.36

Due to complications arising from hierarchical structure of multilevel regression models (Snijders and Bosker; 1994), and to different sample sizes in the models, R^2^ change cannot be interpreted as a relative goodness-of-fit measure across models

**Fig. 3:**
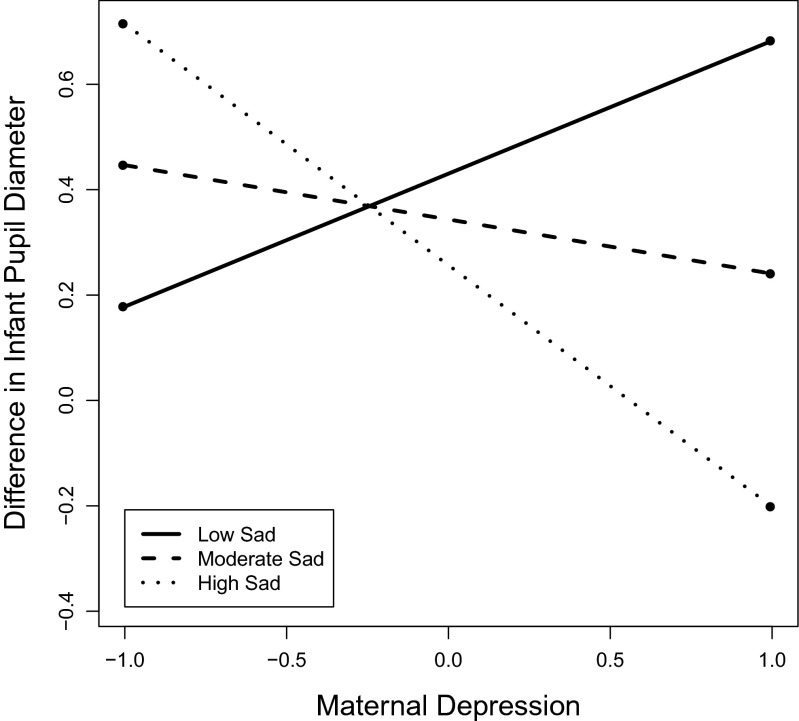
The plot for the association between maternal depression (z-scores on the x-axis) and the change in infants’ pupil response to objects following emotion-object pairing (z-scores on y-axis) at moderate, low and high levels of infants’ sad temperament. The moderate, low and high levels of infant sadness were set to mean, and 1 *SD* below and above the mean respectively. Inspection of regions of significance (i.e., continuously plotted confidence intervals) revealed that the slope of the association was significant for infants with low (*z* < −0.1.37), and high levels of sadness *z* > 0.34), while it was not significant for infants with moderate levels of sadness. The association between infants’ pupil response and mothers’ depression was positive for infants with low levels of infant sadness, while it was negative for infants with high levels of infant sadness

Finally, to explore whether variation in depressive and anxiety symptoms of parents explains infants’ processing of unfamiliar objects, we repeated the same analyses of individual differences first with scores of parental depression, and next of parental anxiety in place of parental negative affect. Paternal and maternal depression (but not anxiety) predicted the difference in infants’ pupil responses to objects after the pairing with emotional expressions. None of the interactions between parental depression and infant temperament were significant, except for the interaction between maternal depression and infants’ sad temperament. The model is presented in Table 6 (*n* = 43^3^). Higher levels of paternal depressive symptoms predicted a more pronounced increase in infants’ pupil responses to objects following the pairing with emotional (vs. neutral) facial expressions. To investigate how the association between maternal depression and infants’ pupil reactivity differed across low, moderate and high levels of infant sad temperament, we inspected the interaction plots and confidence bands (see Fig. 3). The plot of the interaction revealed that the association between maternal depression and infants’ pupil responses was negative for infants high in sad temperament while it was positive for infants with low in sad temperament. The association was not significant for infants with moderate levels of sad temperament. Confidence bands revealed that the association between mothers’ depression and infants’ pupil responses was significant for *z-*values of infant sadness < − 1.37, and > 0.34). Parental anxiety did not significantly predict infants’ pupil responses, neither alone, nor in interaction with emotion or infants’ temperament.

**Table 6:** The associations of infants’ pupil dilation to objects with infants’ negative temperament and with parental depression

Parameter	*B*	*SE*	*p*
Intercept	0.34	0.13	0.010
Happy	−0.22	0.17	0.211
Fearful	−0.22	0.17	0.200
Sad	−0.25	0.17	0.154
Head/gaze direction	0.03	0.09	0.746
Picture time	−0.01	0.00	0.001
Infant fear	0.12	0.09	0.203
Infant sadness	−0.09	0.11	0.435
Maternal depression	−0.10	0.11	0.338
Paternal depression	0.21	0.10	0.036
Infant sadness*maternal depression	−0.36	0.12	0.005
Order	−0.03	0.03	0.413
Block	0.06	0.27	0.815

The neutral face was the reference for emotion effects. *R* ^*2*^= 0.36

To test individual differences in the change in infants’ processing of objects paired with facial expressions, we first added the main effects of infants’ temperament, and of parents’ negative affect to the object model in Table 3. This model is presented in Table 5 (*n* = 46). Among main effects, the effect of maternal (but not paternal) negative affect was significant. Higher levels of maternal negative affect predicted a less pronounced difference in infants’ pupil responses to objects after the pairing with emotional (vs. neutral) facial expressions. None of the tested interactions were significant in this model.

Finally, to explore whether variation in depressive and anxiety symptoms of parents explains infants’ processing of unfamiliar objects, we repeated the same analyses of individual differences first with scores of parental depression, and next of parental anxiety in place of parental negative affect. Paternal and maternal depression (but not anxiety) predicted the difference in infants’ pupil responses to objects after the pairing with emotional expressions. None of the interactions between parental depression and infant temperament were significant, except for the interaction between maternal depression and infants’ sad temperament. The model is presented in Table 6 (*n* = 43). Higher levels of paternal depressive symptoms predicted a more pronounced increase in infants’ pupil responses to objects following the pairing with emotional (vs. neutral) facial expressions. To investigate how the association between maternal depression and infants’ pupil reactivity differed across low, moderate and high levels of infant sad temperament, we inspected the interaction plots and confidence bands. The plot of the interaction (see Fig. 3) revealed that the association between maternal depression and infants’ pupil responses was negative for infants high in sad temperament while it was positive for infants with low in sad temperament. The association was not significant for infants with moderate levels of sad temperament. Confidence bands revealed that the association between mothers’ depression and infants’ pupil responses was significant for z-values of infant sadness < − 1.37, and > 0.34. Parental anxiety did not significantly predict infants’ pupil responses, neither alone, nor in interaction with emotion or infants’ temperament.

## Discussion

The current study examined infants’ attention to emotion and gaze direction as essential skills for infants’ understanding of emotion-object associations in SR situations. We used an eye-tracking paradigm to investigate how 14-to-17-month-old infants’ attention to unfamiliar objects changes after seeing these paired with happy, fearful, and sad (vs. neutral) faces with head and gaze direction towards (vs. away) from the objects. To explore individual differences in infants’ attention, the associations between infants’ attention to emotion and referential cues and parents’ as well as infants’ negative emotional dispositions were tested. Below, we first discuss the findings on infants’ attention to emotion and referential cues, and next the findings concerning individual differences.

Infants showed more attention (larger pupils) to negative faces (fearful and sad) independent of the threat-relevance than neutral faces when presented alone, while they showed the largest increases in attention to objects that were paired with neutral (vs. emotional) faces. The effect of gaze direction was not significant, and the effects of emotion on infants’ attention to objects did not significantly change as a function of gaze direction. Despite several differences in methodology and age group, these results are remarkably similar to those of Hoehl and Striano (2010b) where 9-month-old infants showed increased attention allocation (measured via ERPs) to fearful (vs. neutral) faces independent of gaze direction, while they showed more attention to objects paired with neutral (vs. fearful) faces gazing towards objects. Although the associations between infants’ ERP and pupil dilation remains to be tested in future studies, increased attention allocation to objects paired with static neutral faces observed both in this previous study at 9 months, and in the current study at 15 months can be explained by infants’ sensitivity to ambiguous information in this period (Campos, Thein, and Owen 2003). Because a static neutral face makes it difficult for the infant to process the threat value of the novel object, it may have triggered increased attention. This increase in infants’ attention allocation to neutral faces may mean that less attentional resources would be available for the processing of the objects during face-object pairing, and therefore objects previously paired with neutral (vs. emotional) expressions would be relatively more novel and trigger more attention when presented alone.

In contrast to the findings of Hoehl and Striano (2010b) that revealed more attention to the objects that have been previously paired with neutral (vs. fearful) faces gazing towards (and not away from) objects in 9-month old infants, referential cues (head/gaze direction) did not affect infants’ pupil dilation to objects paired with emotional (vs. neutral) expressions in the current study. Thus, it seems that neutral (vs. emotional) faces triggered enhanced attention to objects, independent of whether they are gazing towards or away from the object. Note that in Hoehl and Striano 2010b, enhanced attention to fearful faces observed during the object-face pairings was also independent of gaze. Considering that infants themselves use looking away as an avoidance strategy in the face of ambiguous SR situations at the end of first year (Aktar et al. 2013; De Rosnay et al. 2006), they may also recognize this avoidant behavior in others. This idea is supported by evidence revealing that later in development, children can form face-object associations in similar experiments even when objects are presented with facial expressions with frontal gaze, (that is, towards the child, and away from objects; Dunne and Askew 2013). Thus, enhanced attention allocation to face-object associations may be less dependent on overt referential cues at the end of first year and beyond.

While it is clear that fearful faces triggered more attention than neutral expressions, the pairing of a neutral face with novel objects did increase infants’ attention to these objects more than a fearful face. Thus, negativity biases in infants’ attention may be specific to faces (and not extend to objects paired with faces). Negativity biases in infants’ attention to negative (vs. neutral) facial expressions were independent of threat-relevance (i.e., it held for both fearful, and sad expressions in the current study). The findings revealing more attention to objects paired with neutral (vs. emotional) expressions are not in line with evidence from SR studies where infants are confronted with ambiguous stimuli like strangers or robot toys (Aktar et al. 2013; Carver and Vaccaro 2007; De Rosnay et al. 2006). In the current study, infants’ attention to referential cues and emotion was measured as components of emotion-object associations in SR situations in a fully computerized task that did not involve direct confrontations with ambiguous objects. Instead, emotion-object associations were presented in the form of static pictures of novel objects and faces appearing on a computer screen. This static presentation may have lowered the functional significance and the threat value of fearful faces, and the overall strength of emotion-object associations. This may explain the lack of an enhanced attention allocation to objects paired with negative emotional expressions. It remains to be investigated whether infants would show a negativity bias in their attention allocation for objects paired with fearful (vs. neutral) facial expressions if their pupil responses were measured during confrontations with these objects in real life.

Concerning individual differences, the results revealed that infants’ temperamental dispositions for sadness, mothers’ negative affect and both parents’ depression are related to physiological correlates of infants’ attention allocation to emotional faces in SR contexts. Higher levels of sad temperamental disposition in infants was linked to larger pupils to emotional (happy, fearful, sad vs. neutral) facial expressions when these were presented alone. This effect did not extend to the processing of the objects paired with these faces. However, in contrast to previous ERP evidence in typically developing infants (De Haan et al. 2004), infants’ fearful temperament was not associated with changes in attention to emotional faces in this study. The decrease in threat value of emotional signals resulting from the lack of a direct consequence of emotion-object associations on infants’ experience may explain why negative faces, and pairing of negative faces with novel objects, did not trigger a different response among temperamentally fearful infants in this study.

Higher levels of maternal negative affect predicted a decrease in the difference in infants’ attention allocation to objects after the pairing with emotional (vs. neutral) facial expressions. Moreover, infants’ sad temperament moderated the associations of mothers’ depression and in infants’ pupil responses to novel objects that were paired with facial expressions. The association of maternal depression and infants’ pupil responses to objects was negative for infants with high levels of sad temperament, while it was positive for infants with low levels of sad temperament and not significant for infants with moderate levels of sad temperament. These findings are in line with the idea that infants’ negative temperamental dispositions may influence the effect of exposure to maternal depression in a non-clinical sample. The negative associations of maternal negative affect and depression with infants’ attention to emotional expressions in the current sample are consistent with previous ERP evidence from clinical samples and non-clinical populations revealing a negative association between infants’ exposure to positive and sad expressions from mothers and their attention to positive and sad expressions in others (De Haan et al. 2004; Field, Diego, and Hernandez-Reif 2009). Note however, that the negative association reported in the current study was in infants’ attention allocation to objects previously paired with positive and negative emotional expressions, and not in their attention allocation to facial expressions. Furthermore, the association of mothers’ depression was found to be positive for infants with low temperamental dispositions for sadness in the current study.

A question that arises from these findings is why infants without sad temperament would show more attention to objects paired with positive and negative facial expressions when their mothers are depressed. In the absence of previous evidence on the interplay between parents’ depression of infants’ sad temperament on infants’ attention allocation and later child outcomes, we can only speculate about the causes and functions of this association. As suggested by Peláez, Virues-Ortega, Field, Amir-Kiaei, and Schnerch (2013), and by Gewirtz and Peláez-Nogueras (1992), increased flat affect and decreased availability of depressed parents may limit infants’ access to mothers’ emotional signals in SR situations, and reduce infants’ frequency of using parents’ emotional signals in SR situations. Enhanced attention to others’ emotional expressions in triadic contexts in case of higher maternal depressive symptoms may be an adaptive response that helps infants gather information in SR situations from others when the depressed mother is not available. Because these infants are not temperamentally likely to respond negatively to novel objects, increased attention to others’ signals may especially be helpful for self-protection against potentially dangerous stimuli in the environment in infants low in sad temperament in this period. Such an increase in attention to others’ facial expressions is also adaptive for infants’ socio-emotional development as it helps to reduce infants’ exposure to depressed mothers’ flat affect. We therefore conclude that enhanced attention to emotional expressions of others may act as a buffer against potential dangers in the environment, and against the effects of exposure to parental depression in infants without sad temperament. The effect seems to be specifically related to mothers’ flat/neutral affect characterizing depression.

The question that arises next is what the function and effect of such a decrease in attention allocation of temperamentally sad infants to objects paired with positive and negative emotional expressions is when the mother has more depressive symptoms. On the one hand, an overall decrease in attention to objects paired with negative facial expressions could protect temperamentally sad infants (who were found to be more vigilant to emotional faces as compared to infants who are not temperamentally sad in this study) against others’ strong negative emotional expressions to ambiguous stimuli. On the other hand, the shutting down to positive emotions could as well be seen as the cost of this early mechanism, which may result in a more flat affect that is less affected by others’ positive emotions in triadic person-infant-object interactions. As high levels of sad temperament is a potential risk for childhood depression, this lowered sensitivity towards positive emotion-object associations is an important finding that needs further study as an early pathway to depressive symptoms in infants of parents with clinical and non-clinical levels of depression.

Higher levels of paternal depression predicted an increase in infants’ attention allocation to objects paired with positive and negative facial expressions. Different from mothers’ depression, the effect of fathers’ depression was independent of infant temperament. Despite significant correlations between paternal negative affect and depressive symptoms, only paternal depression but not negative affect predicted infants’ attention, suggesting that the effect may be specifically related to the flat affect that accompanies depressive symptoms.

The results reveal that depressed moods of mothers and fathers have distinct associations with temperamentally sad infants’ attention allocation to objects in SR contexts. Infants with high sad temperament showed increased attention to objects paired with facial expressions when their father reports higher levels of depression, while they showed decreased attention when their mother reports higher levels of depression. These findings seem to be in line with the idea of distinct roles of mothers and fathers in infants’ socio-emotional development (Bögels and Perotti 2011; Bögels and Phares 2008). Bögels and colleagues explain the differences between mothers’ and fathers’ role in child development based on the differentiation in mother and father domains of expertise throughout the course of evolution. While mothers specialized in care and comfort, fathers specialized in dealing with the external world. According to this theory, fathers may know best whether novel/ambiguous stimuli should be avoided or confronted, and infants know that fathers know best. However, depressed fathers are not able to fulfill this role of showing their infant how to interpret novel stimuli, which may explain why fathers’ but not mothers’ depression is related to increased attention for novel objects paired with others’ emotional faces in the current study. Current findings reveal that exposure to higher levels of paternal depression may increase infants’ attention towards others’ positive and negative emotions. As enhanced attention to negative stimuli characterizes depression and anxiety (Leppänen 2006; Van Bockstaele et al. 2014), exposure to paternal depressed moods may add up to the risk for later psychopathology in infants’ with high levels of sad temperament.

In contrast to depressive symptoms, mothers’ or fathers’ anxiety symptoms did not predict infants’ pupil responses to novel objects. Considering that parents’ fearful/anxious expressions have an observable effect on infants’ reactions in triadic SR contexts (De Rosnay et al. 2006), it is difficult to explain why the variation in parents’ anxiety symptoms did not predict infants’ attention to objects paired with negative faces. Differently from depression that is related to an overall increase in parents’ flat and sad emotional expressions, parents’ anxious expressions only manifest in reaction to specific stimuli, and fade in the absence of these stimuli (except for generalized anxiety, American Psychiatric Association 2013). Thus, infants’ exposure to negative affect may be more prolonged in the case of depression compared to anxiety, resulting in a more easily detected effect in non-clinical samples.

The findings of the current study should be interpreted considering the following limitations. First, although the current study tested the effect of emotion and referential cues on infants’ physiological reactivity in triadic person-infant-object contexts, it remains unknown how infants’ physiological reactivity in the task relates to their behavioral and physiological responses to novelty in real life SR situations. Previous ERP evidence has revealed that parents’ emotion and referential cues towards novel toys observed in real life SR situations influence infants’ subsequent attention allocation to the pictures of these toys presented on a computer screen (Carver and Vaccaro 2007), while further evidence is needed to fully establish the associations between physiological and behavioral indices of infants’ attention to emotion and to referential cues in real-life SR situations and in computerized SR tasks. Second, the current study only included static visual information to test the effects of gaze direction and emotion, while evidence reveals a larger influence of auditory and multimodal cues on infants’ emotion processing (Grossmann 2010), and on behavior in SR contexts in infancy (Mumme, Fernald, and Herrera 1996). Future studies using computerized tasks should consider using dynamic facial expressions with dynamic object displays to explore infants’ emotion processing. Third, although the findings seem to be in line with the idea of distinct roles of mothers and fathers in infants’ socio-emotional development (Bögels and Perotti 2011; Bögels and Phares 2008), the differences in infants’ attention allocation were tested only with female faces in the current study. It remains to be investigated whether these individual differences also hold when infants are tested with female and male faces, as well as with their mothers’ and fathers’ faces. Finally, the cross-sectional and non-experimental design of the current study precludes any prospective or causal inference on the effect of parental negative affect, depression, and anxiety, and of infants’ temperament on infants’ attention to facial expressions, and to novel objects paired with these expressions in triadic emotion learning contexts. Despite these limitations, the current study provides the first evidence on the processing of emotion and referential cues via pupillometry in triadic person-infant-object interactions, and reveals significant associations of infants’ negative temperament and parents’ negative emotions with infants’ pupil reactivity to emotional facial expressions, and to emotion-object associations.

## Footnotes

1 Among 41 infants with eye-tracking data in the Face Alone trials (see Table 3.a), data from 8 infants were missing in the analyses of individual differences because the data on fathers’ negative affect was missing.

2 Among 56 infants with eye-tracking data in the Object Alone trials (see Table 3), data from 10 infants were missing in the analyses of individual differences because the data on fathers’ negative affect was missing.

3 Among 56 infants with eye-tracking data in the Object Alone trials (see Table 3), data from 13 infants were missing in the analyses of individual differences because the data on fathers’ depression was missing.
